# Batch adsorption study in liquid phase under agitation, rotation, and nanobubbles: comparisons in a multi-parametric study

**DOI:** 10.1007/s11356-023-30342-w

**Published:** 2023-10-19

**Authors:** Sofia L. Kouvalakidou, Athanasios Varoutoglou, Khuloud A. Alibrahim, Abdullah N. Alodhayb, Athanasios C. Mitropoulos, George Z. Kyzas

**Affiliations:** 1 Hephaestus Laboratory, Department of Chemistry, International Hellenic University, GR-65404 Kavala, Greece; 2 Department of Chemistry, College of Science, Princess Nourah bint Abdulrahman University, P.O. Box 84428, Riyadh, 11671 Saudi Arabia; 3 Department of Physics and Astronomy, College of Science, King Saud University, Riyadh, 11451 Saudi Arabia

**Keywords:** Adsorption, Rotation, Agitation, Nanobubbles, Dye, Mechanism, Inclination

## Abstract

Concern for environmental protection has increased throughout the years from a global perspective. To date, the predominance of adsorption as treatment technique in environmental chemistry remains unchallenged. Moreover, the scientific attention for investigating nanobubbles due to their unique properties has turned the search for their application in environmental processes with special emphasis on water treatment. This study is aimed at investigating the effect of rotation on batch adsorption process using commercial activated carbon as adsorbent material, compared with the widely used method of agitation. As liquid medium, deionized water and deionized water enhanced with nanobubbles (of air) were used. The wastewater was simulated by dissolving a common dye as model pollutant, methylene blue, at concentration of 300 mg/L in the tested liquid. The results indicated that the utilization of nanobubbles resulted in an improvement on adsorption rate, compared to the corresponding values of deionized water solutions. These results may lead to promising applications in the future, since just 1 h of operation increases the water purification and thus provides a simply applied, cost-effective, and rapid alternative.

## Introduction

**Fig. 1:**
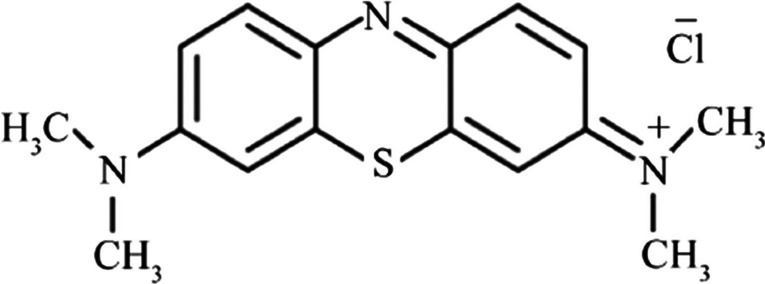
The chemical structure of methylene blue

Since the industrial revolution, the expansion of industrial activities to meet the global population’s demands for material goods has dramatically increased the environmental pollution. Among the different types of environmental pollution, water contamination has posed extreme concern in the last decades. The degradation of aquatic environment from colored wastewater affects the aquatic flora/fauna and all the living beings (Kishor et al. 2021; Katheresan et al. 2018). According to recent works, it is estimated that the amount of dyes produced every year is more than 700.000 tones, of which about 100,000 are discarded without further treatment in the aquatic environment as colored effluents (Nazar De Souza et al. 2021; Tkaczyk et al. 2020). The major industries that produce considerable volume of colored wastewater are textile, dyeing, paper and pulp, tannery and paint, and dye manufacture (Fig. 1). Textile factories release the largest amount of wastewater, contributing more than the half amount of dye effluents observed worldwide (54%) (Kishor et al. 2021).

Synthetic dyes are used extensively in textile industry. Their molecular structure ensures they present great stability and appear poor degradability. Due to specific groups in their molecular structure, such as azo- and nitro- or various aromatic compounds, toxic amines occur after reactions or carcinogen compounds after microbial metabolism respectively (Kyzas and Lazaridis 2009). Even at low concentrations of 1 mg/L, some dyes have high chromaticity, affecting sunlight’s penetration, with negative output in the photosynthesis of underwater plants, obstructing gas solubility. Furthermore, the presence of chemicals in industrial dye wastewater is correlated with high toxicity and, in some cases, carcinogenic and mutagenic effects (Banat et al. 1996; Giannakoudakis et al. 2016). The treatment of industrial dye effluents has become a major concern for environmental scientists as well as an ongoing challenge. Although there have been established plenty of dye removal methods and much more were studied, the most commonly used are based on adsorption (Kyzas et al. 2018).

The application of adsorption for water purification is chosen considering the great removal efficiency, the simplicity of the method, its versatility, and the low-cost, comparing with other traditional or even innovative methods. Activated carbon is the main adsorbent used globally for both industrial and domestic water treatment methods. Its widespread use can be associated to both the extended surface area and the high degree of porosity of different activated carbons present.

One novel modification of traditional water treatment methods that has been studied experimentally in the last decade and shown technological advantages as well as promising application prospects is the addition of nanobubbles (NBs) for water purification. Thanks to their unique physicochemical characteristics, bNBs are applied in a wide range of fields such as wastewater treatment, agriculture, medicine, flotation, and cosmetics. Their rapid prevalence in such a broad spectrum of applications is related to their surface load, the volume they occupy in liquid media as a function of their size, and their extended longevity (Favvas et al. 2021). Last but not least, it would be an omission not to mention that by inserting NBs in an aquatic solution, it has been observed that the time needed for adsorption equilibrium is reduced significantly (Gurung et al. 2016; Kyzas et al. 2019; Kyzas et al. 2020; Temesgen et al. 2017; Wu et al. 2021).

In this work, the comparative removal of a model dye pollutant (methylene blue—Fig. 1) onto commercial activated carbon has been studied, by using either deionized water (DW) or deionized water enriched with atmospheric air bulk nanobubbles (DW-NBs). The selection of air NBs (and not O_2_-NBs or N_2_-NBs) is due to the fact that this type of NBs is generated from both oxygen and nitrogen gas and not separately. So, we can export more realistic conclusions about their application. In the experimental procedure, the effectiveness of a relatively recent method used for batch adsorption, rotation in tube, has been also tested, compared to the conventional method of agitation.

## Theory

### Adsorption dynamics

Numerous sorption systems have been examined in order to analyze the kinetics of adsorption process. The pseudo-first-order rate equation for the adsorption of dissolved molecules from a solution has been widely applied for a long time. This equation is described by the formula:

1 $${\textrm{C}}_{\textrm{t}}={\textrm{C}}_0-\left({\textrm{C}}_0-{\textrm{C}}_{\textrm{e}}\right)\left(1-{\textrm{e}}^{-{\textrm{k}}_1\textrm{t}}\right)$$

where *C*_*e*_, *C*_0_, and *C*_*t*_ are variables describing the concentrations (mg/L) at equilibrium (*e*), the initial (0), and at any time (*t*), respectively, and *k*_1_ is the first-order adsorption rate constant (min^−1^) (Azizian 2004).

On the other hand, another widely used model based on equilibrium adsorption is the pseudo-second-order equation, which is expressed as (Eq. 2):

2 $${\textrm{C}}_{\textrm{t}}={\textrm{C}}_0-\left({\textrm{C}}_0-{\textrm{C}}_{\textrm{e}}\right)\left(1-\frac{1}{1+{\textrm{k}}_2\textrm{t}}\right)$$

where *k*_2_ (mg·g^−1^·min^−1^) is the second-order rate constant (Azizian 2004; Ho and McKay 1999)

Moreover, one of the most critical parameters for the proper understanding of adsorption mechanism is equilibrium time. At this point, the amount of adsorbate molecules onto the adsorbent reaches a constant value. In particular, the concentration of adsorbates onto the adsorbent’s surface is in dynamic equilibrium with the concentration of the dissolved adsorbate molecules in the bulk solution. The time required to reach this state is referred to as adsorption equilibrium, and the number of molecules adsorbed at that moment represents the maximum adsorption capacity in the given operational conditions (Ayawei et al. 2017; Foo and Hameed 2010; Hameed et al. 2007).

Adsorption isotherms reveal the way that molecules are distributed between liquid and solid phases, when the system reaches the equilibrium state, at certain temperature and pH. There are different isotherm models, and each of them expresses a different way of how the solution interacts with the adsorbent. The important step which needs to be made here is fitting of isotherm data in different isotherm models and selection of the most suitable model based on a correlation factor. Over the years, a wide range of isotherm models have been developed. Two of the most commonly used are Langmuir and Freundlich models (Ayawei et al. 2017; Foo and Hameed 2010; Hameed et al. 2007).

Langmuir empirical isotherm model is based on the assumption that the adsorbate is arranged in a monolayer on adsorbent’s surface, on a fixed number of defined adsorption sites, which are identical and equivalent, with no interaction between the adsorbed molecules. Finally, Langmuir isotherm requires homogenous adsorption, where each molecule possesses consistent enthalpies and adsorption activation energy, with no mobility of the adsorbate at the surface level. The Langmuir model is represented by the following equation (Eq. 3) (Langmuir 1918):

3 $${\textrm{Q}}_{\textrm{e}}=\frac{{\textrm{Q}}_{\textrm{m}}{\textrm{K}}_{\textrm{L}}{\textrm{C}}_{\textrm{e}}}{1+{\textrm{K}}_{\textrm{L}}{\textrm{C}}_{\textrm{e}}}$$

where *Q*_*e*_ (mg/g) is the concentration of adsorbate onto adsorbent’s surface, after reaching equilibrium, *C*_*e*_ (mg/L) is the concentration of the adsorbate in the solution, *Q*_max_ (mg/g) is the maximum amount of the adsorbate that can be found in the surface of the adsorbent after equilibrium, and *K*_*L*_ (L/mg) is a Langmuir constant.

The assumptions on which Langmuir equation is based are not always valid for adsorbents with heterogeneous surface. Freundlich empirical model has been proposed to overcome the limitations of mono-layer’s formation and to be applied for uneven adsorption energies at the sites over the surface. The adsorption begins with the occupation of the stronger binding sites and is completed by the exponentially decreasing of the adsorption energies. The well-known Freundlich equation is given below (Eq. 4) (Freundlich 1906):

4 $${Q}_e={K}_F{C}_e^{1/n}$$

where *K*_*F*_ (mg^1−(1/n)^ L^1/n^g^−1^)) is the Freundlich isotherm constant, related to adsorption capacity and n factor refers to adsorption intensity.

### Nanobubbles in brief

In the endeavor of developing a novel water treatment method, there has been a notable trend of using nanobubbles in various conventional technologies. The most common definition of nanobubbles (NBs) describes them as nanoscopic gaseous cavities with diameters less than 1 μm (Favvas et al. 2021; Kyzas et al. 2020). They can exist on a liquid-solid interface (surface NBs) or dispersed in the solution (bulk NBS). Some of the important advantages they appear are the reason of attracting increased attention from researchers and are related to their extraordinary longevity (from weeks to months) as well their unique physicochemical properties (Kyzas and Lazaridis 2009; Michailidi et al. 2020).

According to classical thermodynamics, an inconsistency seems to appear, as it would be expected the NBs to be dissolved immediately according to Young–Laplace Law (Eq. 5):

5 $$\Delta \textrm{P}=\frac{2\gamma }{R}$$

where ∆P = *P*_*vap*_ − *P*_*liq*_ is the difference between the internal and external pressure of the nanobubbles (Laplace pressure), γ is the surface tension of bubble-liquid interface, and *R* is the spherical NB’s radius.

A characteristic of NBs which is of particular interest and is related to their stability over time is their high interface potential caused by the surface charge of NBs. A way to quantify the magnitude of electrostatic repulsion or attraction between particles and NBs is zeta potential. If the total amount of suspended particles has high zeta potential, they remain stable in the solution; otherwise, they are attracted and finally coagulated. In general, the surface of NBs is negatively charged with hydroxyl groups (OH^-^), on which positively charged hydrogen ions (H^+^) are gathered and form a double layer. It is considered that the stability of NBs is partly due to the electrostatic repulsive forces caused when neighboring NBs approach each other, preventing flocculation (Gurung et al. 2016; Hameed et al. 2007).

## Experimental procedure

### Devices, materials, and methods

The adsorbent used for this experiment was commercial activated carbon (bis(2-ethylhexyl)phosphate modified) from Sigma Aldrich, without further treatment, while the model dye pollutant (methylene blue, MW=319.86 g/mol, purity=99%, which has been taken into consideration in all calculations) was supplied by Panreac. For adjusting the solution pH before the treatment, hydrochloric acid and sodium hydroxide from Sigma Aldrich were used, diluted until 0.01 and 0.1 mol/L. For the needs of the experiment after treatment, the solution was filtrated by nylon membrane filters with a porous diameter of 0.45 μm. The concentration of the filtered solution was measured by a double-beam UV-Vis spectrophotometer (model U-2900, Hitachi).

#### Rotation device

**Fig. 2:**
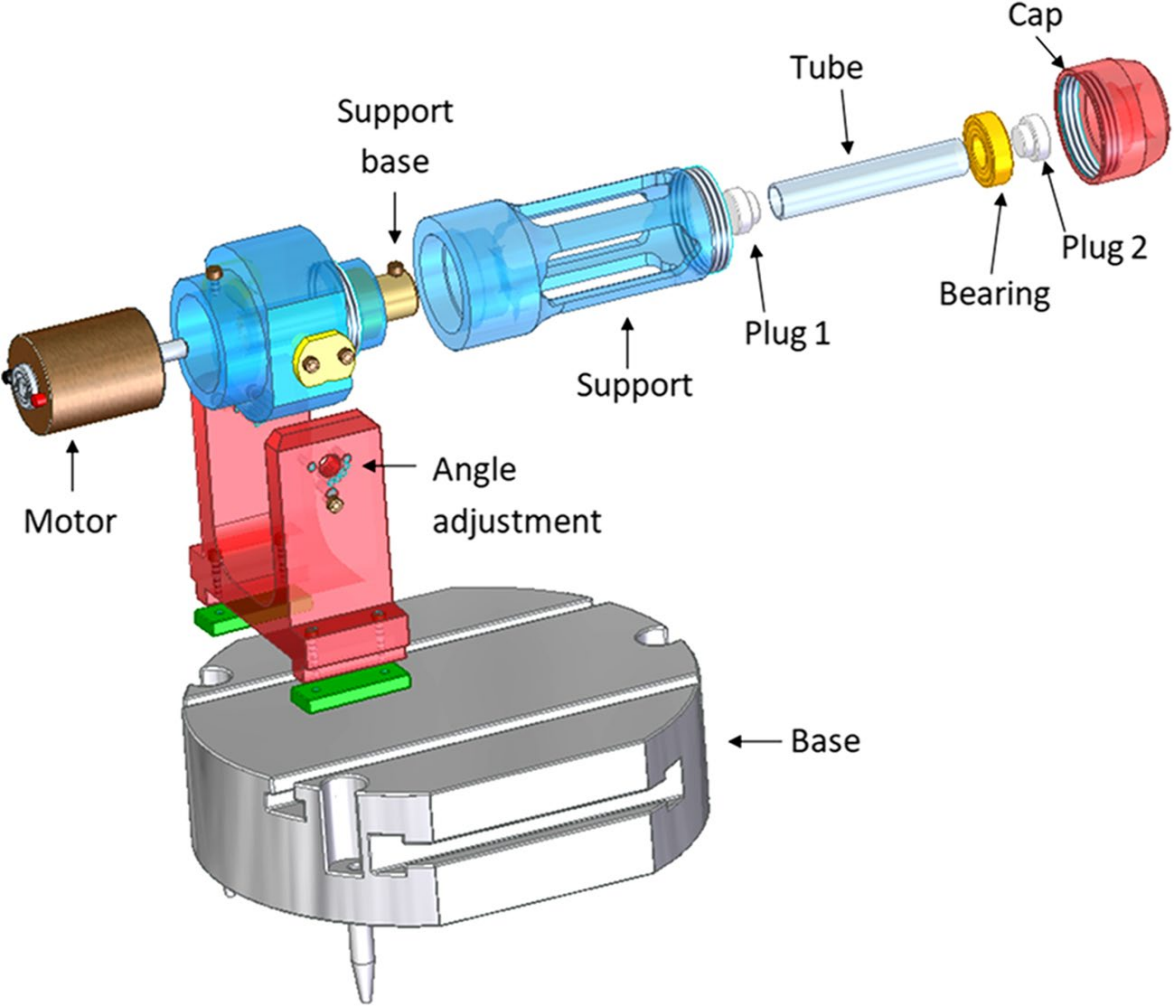
Illustration of the rotation device

For the needs of the current work, a device was constructed allowing rotation and angle adjustment (Fig. 2).

The device is located on a metallic base, followed by a predefined angle adjustment area (0, 25, 45, 60, and 90 degrees). The whole equipment is powered by an adjustable voltage power supply, connected directly to the motor which triggers rotation. Α glass tube, designed for inserting the solution intended for treatment, is placed in a metallic cylinder which provides protection during the whole process. This part of the device is screwed on a support base, connecting all the components of the device. A metallic bearing is placed around the tube’s plug to facilitate the rotational move. Finally, a metallic cap is screwed at the end of the device to ensure the stability of the system.

#### Nanobubbles’ production

Deionized water enhanced with bulk air nanobubbles (DW-NBs) was produced for the needs of these experiments. The formation of nanobubbles can be induced in several ways, depending on various internal or extrinsic factors, such as the medium and surface characteristics or intense shaking and cavitation respectively (Demangeat 2015; Wu et al. 2012). In the current work, the DW-NBs were produced by a NB generator taking advantage of a Venturi tube, causing hydrodynamic cavitation (Michailidi et al. 2020; Mitropoulos and Bomis 2016). At the time a gas-liquid solution flows through the Venturi tube, bubbles are formed as a result of the reduction and subsequent raising of the pressure. The pressure inside the Venturi tube can be decreased by increasing the liquid’s velocity in the conical convergent zone of the tube due to the narrow diameter. In this generator, in addition to hydrodynamic cavitation, the rough surface characteristics are utilized for affecting the fluid flow and converting the system from gas-liquid solution into a colloidal phase.

**Fig. 3:**
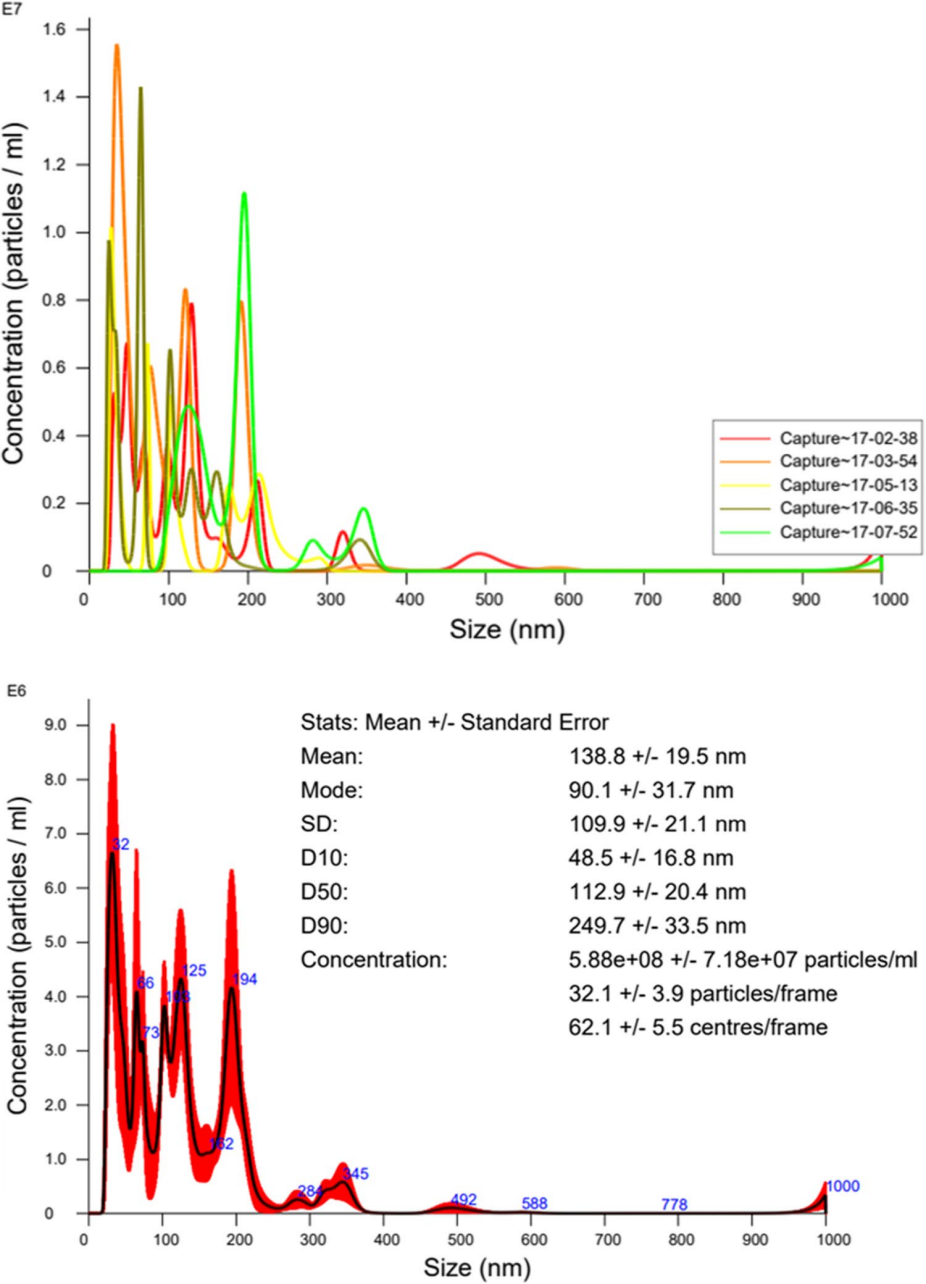
Characteristics of the produced air NBs through NTA analysis

The DW-NBs were produced by adding 4 L of deionized water into the generator’s vessel, while the gas phase (atmospheric air) was filtered for removing traces of suspended nanoparticles, before infusing to the water tank. The operating time to produce the bulk NBs was thirty minutes and the generator’s pressure was about 3.5 bars. The average size of air NBs was in the range of 138.8 nm approximately and their concentration in the solution was about 5.88 × 10^8^ particles/mL. For measuring these values, NTA (nanoparticle tracking analysis) analysis took place, using NanoSight LM10 by Malvern Panalytical, which utilizes the Brownian motion of the nanoparticles to define the size and concentration of nanobubbles (Fig. 3) (Foudas et al. 2023).

The *z*-potential of the air-NB enriched water sample was measured. The value of −8.8±1 mV implies a negatively charged surface. *Z*-potential, an electrokinetic potential at the shear plane, describes the interaction of a solid-liquid interface (where the liquid velocity is zero) at the boundary between the compact layer and the diffuse layer, and it is clear that it is defined to the dispersed phase-particles-conformation into the polar solvent. Zeta potential is a key parameter in determining the interaction energy between particles and hence the stability of colloid suspension systems. In our system, the role of “nanoparticles” plays the nanobubbles which are dispersed into the water bulk phase (Li 2004). This value is close to what is also reported for *z*-potential of bubbles in pure water (Hammadi et al. 2016).

### Adsorption experiments

The experiments were carried out in a rotating tube under the effect of centrifugal force and in shaking bath. For both techniques, deionized water was used as liquid phase, but in the second scenario, the deionized water was enhanced with environmental air NBs. The parameters studied were the effect of pH, contact time, and initial concentration for both rotation and agitation. Further experiments were conducted for the rotational motion, in order to examine the impact of number of rounds per minute, the inclination angle (inclination of the device is the angle between the device and the positive direction of *x*-axis. The value of this angle is between 0 and 180°), and the mass of adsorbent in adsorption effectiveness.

#### Effect of pH on adsorption

In order to study the effect of pH on the adsorption of MB by activated carbon, dye solutions were prepared, whose pH was adjusted to the desired values (2, 4, 6, 8, 10, and 12—the whole pH range was selected to be studied (from strongly acidic to strongly alkaline) so as to simulate all the possible pH conditions of effluents); so after fixing the pH value, the initial concentration of the solution is 300 mg/L. Specifically, a stock solution with 1,000 mg/L dye concentration was prepared from which 3 mL was placed in a beaker, diluted and modified as it concerns the pH value, by adding sodium hydroxide (0.01 and 0.1 M) or hydrochloric acid (0.01 and 0.1 M) up to 10 mL which was the desired volume. Dilution was performed with deionized water in the first case and with deionized water enhanced with air bNBs in the second. The mass of adsorbent used was 0.02 g. The produced solution was added in the glass tube and subjected to rotation for 3 h at 240 rpm. At the end of three hours, the samples are extracted using a syringe and filtered through a nylon membrane filter of 0.45 μm pore size. Their final concentration was determined by monitoring the absorbance of dye solution by a double-beam UV-Vis spectrophotometer (model U-2900, Hitachi).

#### Effect of contact time on adsorption

In order to study the effect of contact time between the adsorbent and the dye solution, experiments are carried out on both rotation device and shaking bath (agitation), in an attempt to evaluate the comparative results between the two techniques. In both cases, the same solution preparation procedure was followed. In particular, 3 mL of the stock dye solution is diluted and adjusted with HCl or NaOH until the optimum pH value, by the final volume of 10 mL, for the production of a 300 mg/L solution. The dilution in these series of experiments also took place with deionized water, and air nanobubble-enhanced deionized water. The amount of adsorbent added was 0.02 g. The contact time studied started every two minutes for the first ten minutes, every five minutes for the first hour, and then at 90, 120, and 180 min. The samples after treatment were filtered through a nylon membrane filter in order to remove the activated carbon, and the final concentration was determined by UV-Vis spectrophotometer.

#### Effect of initial concentration on adsorption

To evaluate the impact of dye’s initial concentration in the solution on adsorption process, methylene blue solutions with deionized water and air bNBs of initial concentrations of 100, 150, 200, 250, 300, 350, 400, 450, and 500 mg/L were prepared. The sample preparation started by diluting the corresponding amount of the stock solution (1–5 mL) with concentration of 1,000 mg/L and adjusting the pH to the optimum value. The mass of activated carbon was 0.02 g. At the end of the procedure, the samples were filtered by the nylon membrane filters and the final concentration was measured by UV-Vis spectrophotometer.

#### Effect of rotation rate

The number of rounds per minute was measured using a tachometer, after filling the glass tube with 10 mL of solution. The voltage applied to the system by the power supply was increased; hence, it caused the raising of rounds per minute. The tested rotational speeds were 240, 520, 820, 1,180, 1,500, and 2,035 rpm; the latter were fixed values of the device. Each sample is produced with concentration of 300 mg/L and adsorbent mass of 0.02 g. The pH and time for the procedure were determined by the optimum pH value and adsorption equilibrium which occurred from the previous experiments, respectively. Here too, the experiment was conducted on the same conditions by adding deionized water and air bNB solution.

#### Effect of inclination angle

As mentioned in the device description area, the device was designed with flexibility as it concerns the selection of inclination angle. The angles chosen for studying the effect of adsorption were 0, 25, 45, 60, and 90 degrees. The solutions are prepared according to the procedure described above, with initial concentration before treatment 300 mg/L and under the optimal conditions (pH and contact time). After filtration, the concentration of the treated samples was calculated through a UV-Vis spectrophotometer.

#### Effect of mass of adsorbent

While keeping the initial concentration stable at 300 mg/L and using the optimal conditions obtained from the previous experiments, the impact of adsorbent’s mass was estimated with weighting and adding in the solution 0.005, 0.01, 0.015, 0.02, 0.025, and 0.03 g of activated carbon. The dye solutions prepared by using both DW and DW are enhanced with air bNBs. The final concentration is measured after solution’s filtration, with UV-Vis spectrophotometer.

## Results and discussion

### Effect of pH and adsorption mechanism

**Fig. 4:**
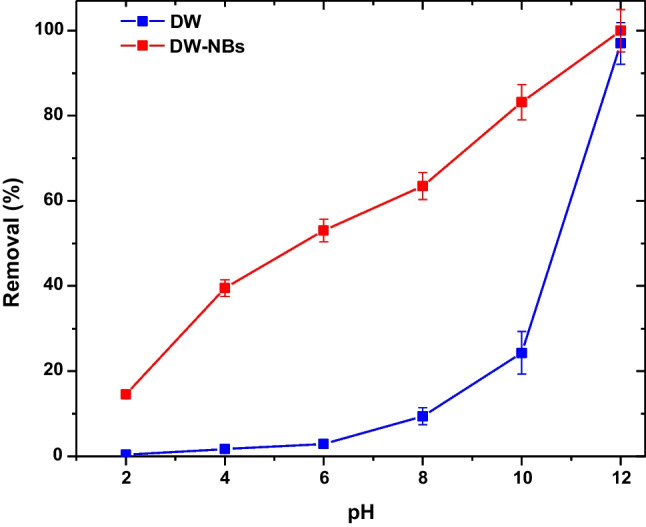
Effect of pH on adsorption by using DW and DW-NBs

A series of experiments are conducted to evaluate the adsorption behavior of the studied activated carbon, in deionized water solution (case 1) and with the addition of air bNBs (case 2), under various pH values (2, 4, 6, 8, 10, and 12). Figure 4 illustrates a diagram of the obtained values. The optimum pH for both cases was 12. In acidic pH values, the competitive reaction of free hydrogen ions and the cationic dye with the remaining negatively charged groups of the adsorbent leads to a critical decrease in the removal rate of ΜΒ. The latter can be useful because apart from the textile wastewaters of acidic pH conditions, there are textile wastewaters (based on cationic dye effluents) which are often highly alkaline (pH 9.0–13.0, as the fixation of dye to fabric requires high pH), highly saline (salinity 3.5–20%, containing Na^+^, Ca^2+^, Mg^2+^, etc.) along with high color, COD, and BOD content (Bhattacharya et al. 2017; Maurya et al. 2006). Also, it should be noted that there are many case studies employing extremely high alkaline real dyeing effluents that need to be treated (Islam and Mostafa 2019; Desai and Kore 2011).

By increasing the concentration of hydroxide ions in the solution, negatively charged groups are created at the surface of the adsorbent, which attract the cationic dye, and therefore, the dye removal rate is improved (Qu et al. 2019). By enriching deionized water with nanobubbles (DW-NBs), a significant improvement in dye removal is noticed over the whole range of studied pH values. In particular, if dye removal is not taken into account at the optimum pH value, 12, where the difference between DW and DW-NBs solution is negligible, the average difference on MB removal between DW-NBs and DW solution over the entire pH range is approximately 45%. This could be explained by the negative charge on the nanobubbles’ surface, assisting the dye’s transfer to adsorbent’s functional groups, as already observed in a previous work (Kyzas et al. 2019)

To understand the adsorption mechanism in line with the nanobubbles effect, it is fact that the bulk liquid effects are precluded since the volume fraction of nanoparticles is extremely small (a simple calculation shows a value less than 10^−6^). But, NBs have been reported to assist significantly the efficiency of flotation technology as wastewater treatment (Temesgen et al. 2017). So, it can be stated that NBs preserve pollutant particles in suspension increasing this way the probability of pollutant/adsorbent contact. Therefore, the effect of NBs should be related to intraparticle processes and the intensification of NB concentration in the activated carbon particle (Kyzas et al. 2019). To further understand, it seems that the most probable scenario is the penetration of the NBs in the pore network and their attachment to the pore walls, but the penetration degree depends on both the size distribution of the NBs and pore size distribution. The contact angle of bubbles should be relatively high in order to give a combined effect of large number concentration of attached bubbles (which can explain the adsorption kinetics acceleration as it will be shown in the following) and a small surface coverage (which is compatible to the small effect of bubbles on the adsorption isotherm).

A normal question to answer is now how the NB diffusion towards particle interior increases the kinetics of solute diffusion. Nanobubbles in water acquire negative surface charges (Yurchenko et al. 2016; Zhang and Seddon 2016). This means that a so-called double layer formed around the NBs consists of counterions and compensates the surface charge to neutralize the whole bubble-double layer system (Israelachvili 2011). The presence of double layer leads to repulsive forces between NBs which are responsible for the stability of NBs against coalescence according to the theory suggested by Boris Derjaguin and Lev Landau, Evert Verwey and Theodoor Overbeek (DLVO theory) (Verwey and Overbeek 1948). This theory explains the aggregation of aqueous dispersions quantitatively and describes the force between charged surfaces interacting through a liquid medium. It combines the effects of the van der Waals attraction and the electrostatic repulsion due to the so-called double layer of counterions. The electrostatic part of the DLVO interaction is computed in the mean field approximation in the limit of low surface potentials—that is when the potential energy of an elementary charge on the surface is much smaller than the thermal energy scale. The existence of double layer decreases the diffusion coefficient of the bubbles, but it is only of the order of 10% as it has been shown by detailed computations (Van De Ven 1989).

In the present study, the dye molecules are positively charged, so attractive forces occur among positively charged dye molecules and negatively charged NBs:

$$\text{NB}-\text{OH}^-\cdot\cdot\cdot\cdot\text{Dye}^+\leftrightarrow\text{NB}-\text{OH}^-\text{Dye}^+$$

The surface pH of activated carbon is 2.9 (meaning that is negatively charged), and the solid adsorbent material can easily attract the positively charged double layer of NBs, which is covered with positively charged dye molecules.

$$\text{NB}-\text{OH}^-\cdot\cdot\cdot\cdot\text{Dye}^+\leftrightarrow\text{NB}-\text{O}\text{H}^-\text{Dye}^+$$

$$\text{NB}-\text{OH}^-\text{Dye}^+\cdot\cdot\cdot\cdot^-\text{ACP}\leftrightarrow\text{NB}-\text{O}\text{H}^-\text{Dye}^+\text{ACP}^-$$

The diffusion coefficient of the NB is smaller than the one of the solute molecules, but this is compensated by the large number of dye molecules carried in the double layer around each NB. So similarly, as in previous works, it is suggested that NBs act as carriers/busses and transfer the dye molecules into the outer porous surface/walls (this can be due to thermodynamic preference). Sometimes, NBs may be penetrated into the porous network because the dimension of each NB is not constant. The above view seems to be the most possible explanation for the influence of nanobubbles on adsorption kinetics combined to a very small influence on adsorption isotherm.

### Effect of contact time-kinetics

**Fig. 5:**
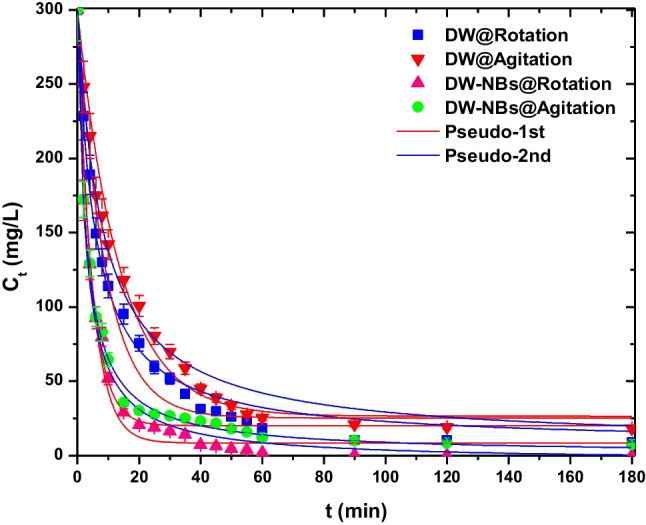
Effect of contact time on adsorption

A graphical representation of the measured final dye concentration over contact time is illustrated in Fig. 5. The four cases represented in the chart are dye solution with deionized water under rotation (DW@Rotation) (case 1), dye solution with deionized water under agitation (DW@Agitation) (case 2), dye solution with deionized water enriched with bulk air NBs under rotation (DW-NBs@Rotation) (case 3), and dye solution with deionized water enriched with bulk air NBs under agitation (DW-NBs@Agitation) (case 4). As it can be seen, the effect of time for all the samples can be divided into three main phases: (i) sharp removal of MB molecules when they come into contact with adsorbent’s surface; (ii) the gradual adsorption phase, when the saturation of activated carbon’s pores has begun; and (iii) the adsorption equilibrium stage, when the process is completed. For all the cases, it was found that adsorption equilibrium is reached after 1 h of process. As can be observed in Fig. 5 as well as the k factors presented in Table 1, the adsorption rate of the pollutant was higher in the solution containing nanobubbles than when plain deionized water was used. For all the studied cases, the samples follow the pseudo-second-order kinetic model. The corresponding parameters are presented in Table 1.

**Table 1:** Kinetic parameters for the adsorption of methylene blue in DW and DW-NB solutions on activated carbon under rotation and agitation

	Pseudo-first order			Pseudo-second order		
	*Q*_*e*_ (mg/g)	*k*_1_ (min^−1^)	*R*^2^	*Q*_*e*_ (mg/g)	*k*_2_ (mg g^−1^ min^−1^)	*R*^2^
DW@Rotation	137.299	0.111	0.976	146.246	0.180	0.989
DW@Agitation	136.756	0.073	0.989	146.206	0.124	0.997
DW-NBs@Rotation	145.769	0.224	0.985	149.932	0.364	0.994
DW-NBs@Agitation	139.938	0.213	0.981	149.525	0.345	0.997

### Effect of rotation speed

**Fig. 6:**
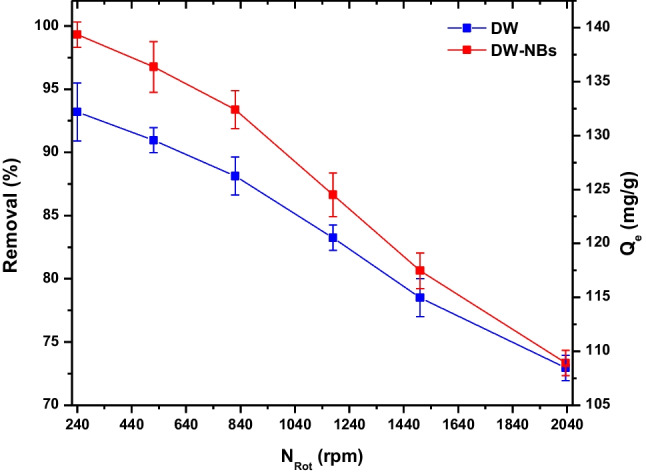
Effect of rotation on adsorption

For the rotational motion, the number of rpm is an important parameter. Thus, the number of rounds per minute (N_Rot_) and its correlation with the dye adsorption were measured (Fig. 6). The optimum rotational speed is found to be 240 rpm with removal 93.19% for DW and 99.32% for DW-NBs. For higher rotational speeds, the adsorbent tends to approach and stick to the tube’s walls and therefore the active sites are narrowed. Hence, the adsorption rate begins to decrease, leading to a reduction in dye removal by 26% and 20% for 2035 rpm, for DW-NBs and DW treatment, respectively, compared to 240 rpm. Furthermore, by increasing the rotational speed, the limitation of the beneficial effect of the nanobubbles was observed, resulting in dye removal tending to that of the deionized water solution.

### Effect of initial concentration (isotherms)

**Fig. 7:**
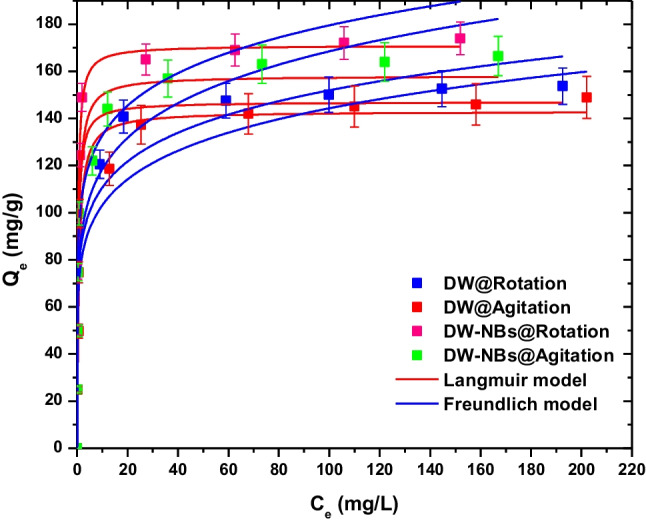
Effect of dye initial concentration on equilibrium—isotherms

The influence of initial methylene blue concentration on adsorption efficiency was studied using the optimum pH and time, with the same mass of adsorbent. The concentrations tested were 100, 150, 200, 250, 300, 350, 400, 450, and 500 mg/L for one hour of operation. Typical adsorption isotherms were prepared for both rotation and agitation, with deionized water and deionized water reinforced with air bNBs. The isotherm models used were Freundlich and Langmuir. From the experimental data, it was obtained that an increase in initial dye’s concentration led to higher amounts of methylene blue sorption. Furthermore, the values of correlation coefficient (*R*^2^), which is a measure for fitting efficiency, indicated that the experimental equilibrium data were consistent with Langmuir equation (Fig. 7). Following Langmuir equation assumptions, the formation of monolayer coverage of dye molecules on adsorbent’s surface is concluded for the current samples (Dada et al. 2012; Foo and Hameed 2010). The fitting parameters are listed in Table 2 for both of Langmuir and Freundlich isotherm models.

**Table 2:** Equilibrium parameters of Langmuir and Freundlich models for the adsorption of MB onto activated carbon (with DW and DW-NBs) under rotation and agitation

	Langmuir equation			Freundlich equation		
	*Q*_*m*_	*K*_*L*_	*R*^2^	*K*_*F*_	*n*	*R*^2^
	mg/g	L/mg		mg^1−(1/n)^L^1/n^ g^−1^		
DW@Rotation	157.117	2.032	0.977	90.461	8.515	0.927
DW@Agitation	146.131	1.514	0.960	82.411	8.255	0.922
DW-NBs@Rotation	172.463	3.051	0.998	104.786	8.906	0.896
DW-NBs@Agitation	161.571	1.909	0.988	90.374	7.557	0.939

### Effect of inclination angle

**Fig. 8:**
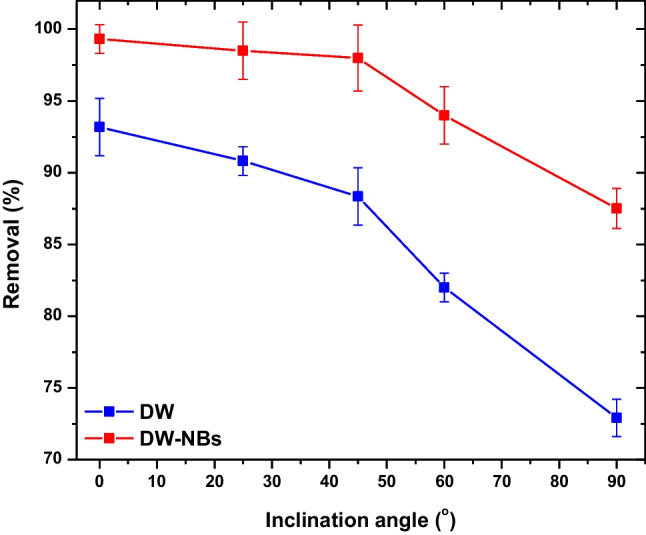
Effect of inclination angle in adsorption efficiency with deionized water and deionized water enhanced with air bNBs

All the previous experiments were conducted for 0 degrees of inclination. However, the impact of inclination angle on adsorption efficiency is important and should be investigated. Thus, a series of experiments are carried out, using the optimal measured values (pH and equilibrium time), modifying the inclination angle into 25, 45, 60, and 95 degrees. The measured values are presented in Fig. 8. By placing the tube into inclination angle of 25 and 45 degrees, a slight reduction in dye adsorption occurred. The optimum inclination was obtained at 0 degrees, while for inclination angle greater than 60 degrees, at 60 and 90°, the removal of dye decreased dramatically.

### Effect of adsorbent’s mass

**Fig. 9:**
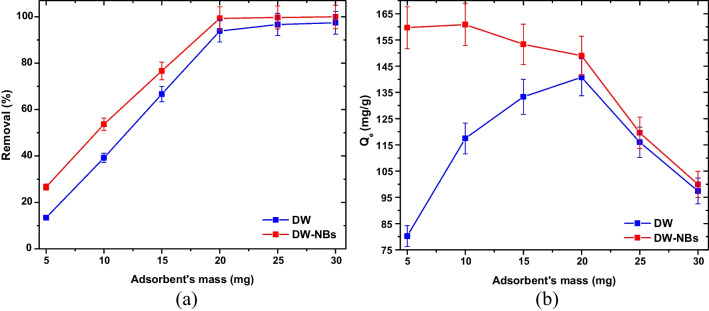
Effect of adsorbent’s mass on **a** dye removal efficiency and **b** adsorption capacity

Finally, the effect of adsorbent’s mass on adsorption efficiency was studied. The adsorbent mass added to the solution was 0.005, 0.01, 0.015, 0.02, 0.025, and 0.03 g, while all the other parameters were kept at constant values. As the adsorbent’s dosage increased, the removal efficiency increased as well, reaching almost 100% of dye removal. The corresponding graphs that occurred from the experimental data are illustrated in Fig. 9a, b, including the effect of adsorbent dose on dye removal efficiency and adsorption capacity, respectively. Τhe optimum adsorption capacity was achieved for 0.02 g of sorbent, while further increasing the mass did not lead to a significant increase in dye removal. The beneficial contribution of nanobubbles on adsorption is observed in Fig. 9b, where at the corresponding adsorbent’s mass values in the absence of nanobubbles, the adsorption capacity of activated carbon is significantly reduced.

## Conclusions

In this work, the adsorption efficiency of methylene blue by activated carbon was studied using deionized water and deionized water enhanced with bulk air nanobubbles. For both cases, the dye solutions are treated under rotation and agitation. The optimum pH for the sorption under all the examined cases was 12. The presence of nanobubbles in the solution resulted in significantly improved results in terms of adsorption of methylene blue on the surface of activated carbon over the entire pH range. After one hour of treatment, equilibrium was reached, with the NBs enriched solutions contributing significantly to reaching equilibrium faster than the corresponding deionized water samples. For all the studied cases, pseudo-second-order kinetics fitted better with the experimental results, indicating that chemisorption is the rate-determining step. After applying Langmuir and Freundlich isotherm models, the correlation factor indicated successfully fitting to Langmuir isotherm, indicating the monolayer coverage of sorbent’s surface at equivalent and fixed adsorption sites. Further experiments were carried out to investigate the rotation-correlated parameters on adsorption, from which it was concluded that the optimum conditions for rotational move were at 0 degrees inclination angle and 240 rounds per minute. The results obtained in the current work are encouraging enough to give rise for further research on the utilization of nanobubbles in wastewater treatment systems and overcoming the ever-increasing barriers to effective water purification.

## Data Availability

Not applicable.
